# Benthic Diatom Blooms of Blue *Haslea* spp. in the Mediterranean Sea

**DOI:** 10.3390/md21110583

**Published:** 2023-11-08

**Authors:** Julie Seveno, Ana Car, Damien Sirjacobs, Lovina Fullgrabe, Iris Dupčić Radić, Pierre Lejeune, Vincent Leignel, Jean-Luc Mouget

**Affiliations:** 1 BIOSSE Laboratory, Le Mans University, 72000 Le Mans, France; 2 Station de Recherches Sous-Marines et Océanographiques STARESO, 20260 Calvi, Francep.lejeune@stareso.corsica (P.L.); 3 Institute for Marine and Coastal Research, University of Dubrovnik, Kneza Damjana Jude 12, 20000 Dubrovnik, Croatia; ana.car@unidu.hr (A.C.);; 4 InBioS–PhytoSYSTEMS Laboratory, University of Liège, B-4000 Liège, Belgium

**Keywords:** bloom, diatom, *Haslea*, marennine, Mediterranean Sea

## Abstract

Blue *Haslea* species are marine benthic pennate diatoms able to synthesize a blue-green water-soluble pigment, like marennine produced by *H. ostrearia* Simonsen. New species of *Haslea* synthetizing blue pigments were recently described (*H. karadagensis*, *H. nusantara*, *H. provincialis* and *H. silbo*). Their marennine-like pigments have allelopathic, antioxidative, antiviral and antibacterial properties, which have been demonstrated in laboratory conditions. Marennine is also responsible for the greening of oysters, for example, in the Marennes Oléron area (France), a phenomenon that has economical and patrimonial values. While blue *Haslea* spp. blooms have been episodically observed in natural environments (e.g., France, Croatia, USA), their dynamics have only been investigated in oyster ponds. This work is the first description of blue *Haslea* spp. benthic blooms that develop in open environments on the periphyton, covering turf and some macroalgae-like *Padina*. Different sites were monitored in the Mediterranean Sea (Corsica, France and Croatia) and two different blue *Haslea* species involved in these blooms were identified: *H. ostrearia* and *H. provincialis*. A non-blue *Haslea* species was also occasionally encountered. The benthic blooms of blue *Haslea* followed the phytoplankton spring bloom and occurred in shallow calm waters, possibly indicating a prominent role of light to initiate the blooms. In the absence of very strong winds and water currents that can possibly disaggregate the blue biofilm, the end of blooms coincided with the warming of the upper water masses, which might be profitable for other microorganisms and ultimately lead to a shift in the biofilm community.

## 1. Introduction

The blooms of microalgae are characterized by a rapid increase in cell density, usually from a single species that outcompetes other microalgae. Benthic blue *Haslea* blooms have been known to occur since the 19th century in oyster ponds; along the northwest Atlantic coast in France [1]. The emblematic species *Haslea ostrearia* (Gaillon) Simonsen, produces marennine, a water-soluble blue pigment. Marennine accumulates in cell apices (intracellular form) and is released in the environment (extracellular form). When *Haslea* spp. blooms occur in oyster ponds, extracellular marennine concentration in seawater increases. While oysters are filtrating seawater to feed on microalgae, they mainly accumulate extracellular marennine on their gills’ mucocytes, which turn green [2]. The color spectrum of marennine solution changes with the pH, from blue in acidic conditions (as in cell apices) to green in more basic conditions (as seawater) [3]. Gastronomically, green-gilled oysters have been considered as a delicacy, which increases their commercial value. Therefore, the first records of blue *Haslea* blooms can be deduced from the description of the greening of oysters in: Colchester Bay, England [4]; in Marennes Oléron, France [5]; in Denmark [6]; in Virginia, USA [7] and in Tasmania, Australia (Hallegraeff, personal communication). On the French west coast, where bivalves are produced, oyster ponds are shallow, semi-closed and dynamic environments, with important daily and seasonal variations of salinity and temperature. They are supplied with both high tides (when the coefficient is above 80–90) and fresh water (heavy rain, rivers). When the flow is weak, the turbidity decreases and leads to shallow ponds with clear water conditions that favor the formation of blue *Haslea* blooms and oyster greening [8]. Green gills have also been observed in other bivalves such as scallops, mussels and clams, suggesting that the greening is a common phenomenon. Moreover, a green coloration can also occur in other animals such as anemones, polychaetes and crabs [1,9].

Blooms or patches of blue *Haslea* spp. have also been observed in open environments, but their dynamics and impact have not been studied yet. For instance, Sauvageau [10] observed “blue diatoms” forming small blue patches on *Colpomenia sinuosa* in the Mediterranean Sea. In Corsica, *Haslea* spp. blooms have been regularly observed with varying intensity in springtime since 1996 (Demoulin, personal communication). On the Dalmatian coast in Croatia, blue *Haslea* spp. blooms were regularly observed in summer months, but the species involved were not identified ([11], Y. Hardivillier and J. Séveno’s personal observations). It is noteworthy that in the Arabian Sea in March 2011, a planktonic bloom of the non-blue species, *Haslea gretharum* Simonsen, has been observed [12].

The link between environmental parameters and organisms is more complex in open ecosystems. Anthropogenic nutrient enrichment results in coastal water eutrophication, leading to an increase in bloom frequency, including harmful algal blooms (HABs) [13]. Climate change, especially warming, may modulate such a phenomenon by affecting the structure of the water column and climatic events. However, blue *Haslea* blooms are not classified as HABs because no high mortality rate has been recorded for organisms in ponds or in open environments, and green-gilled seafood has been cleared for human consumption. Nevertheless, their impacts on microenvironments have yet to be unraveled.

Studies in laboratory conditions provided additional clues on bloom dynamics by examining how *Haslea* spp. cultures were affected by changes in abiotic parameters. Studies on *H. ostrearia* cultures demonstrated that this species is euryhaline [14] and is able to acclimate well to high irradiance and UV [15,16,17]. They also demonstrated that extracellular marennine concentration depends more on light quantity and quality than on cell density and nutrient status [18,19,20]. Nevertheless, as silica is recycled slower than nitrogen or phosphorus [21], it is often one of the limiting nutrients and, thus, the supplement of silica could favor *H. ostrearia* multiplication and marennine production.

The genus *Haslea* encompasses a total of 40 species [22]. Although *Haslea* belongs to the top 20 diatom genera observed in the different oceanic provinces, in particular being widely distributed in the South Pacific Ocean [23], little is known about blue *Haslea* blooms out of the context of oyster greening. Before the now widespread use of genetic markers to identify species, all observed blue *Haslea* cells were attributed to *H. ostrearia*. In addition to *H. ostrearia*, there are currently four other species of blue *Haslea*: (1) *Haslea karadagensis* Davidovich, Gastineau and Mouget; (2) *Haslea provincialis* Gastineau, Hansen and Mouget; (3) *Haslea nusantara* Mouget, Gastineau and Syakti; and (4) *Haslea silbo* Gastineau, Hansen and Mouget. More recently, the presence of blooms occurring in the marshes of the North Carolina coast (USA) was related to the presence of the species *H. silbo* [24]. Thus, to date, only *H. ostrearia* and *H. silbo* have been known to form benthic blooms, in oyster ponds or open environments. Microalgae spores are known to be an important factor initiating blooms [25]; however, the formation of spores by *Haslea* spp. has not been observed either in laboratory or in natural environment.

Therefore, the objective of this research was to describe benthic blooms of blue *Haslea* spp. in the Mediterranean Sea, using descriptive, genetic and statistical analyses. As benthic microalgae, the term bloom for blue *Haslea* spp. is used when the density of cells is high enough to see blue patches on different substrates by the naked eye.

## 2. Results

### 2.1. Corsica (France)

**Figure 1:**
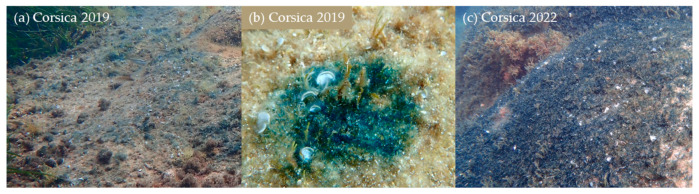
Bloom patches with different blue intensities: (**a**) light, (**b**) medium, (**c**) high.

**Figure 2:**
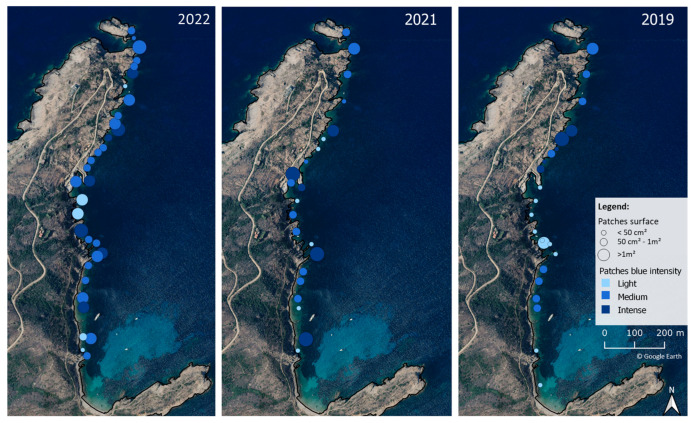
Observations of blue *Haslea* blooms along the Revellata Bay, Corsica, in spring 2019, 2021 and 2022. The blue intensity (see legend) refers to the density of *Haslea* spp. population, as illustrated in Figure 1.

The development of blooms was studied over a three-year period (2019–2022) during which, blue patches of different color intensities (Figure 1) were observed on various substrates. The surface of blue patches varied from a few square centimeters to several square meters. The sites with maximum density (vast surface and high blue intensity) changed over the years (Figure 2). Blooms covered more areas and with a higher cell density in 2022 than in 2021 and 2019.

In order to estimate the distribution of the bloom, the relationship between the intensity of the blue patches and the density of *Haslea* cells present in the patches was first evaluated. When there were no visible blue patches, *Haslea* cells represented, on average, 1% of the total diatom community. When blue patches were visible, *Haslea* cells represented, on average, 24.6% of the total diatom community (Table 1).

There were significant differences (*t*-test: *p*-value < 0.05) between the percentage of *Haslea* spp. in the diatom community and the intensity of the blue color of patches. For samples with patches presenting a very high blue intensity (Figure 1), *Haslea* spp. counted for an average of 43% of the whole diatom community (Table 1) and a maximum of 80% has been observed in one sample. There were no significant differences (*t*-test: *p*-value > 0.05) in the representation of *Haslea* spp. between *Padina* sp. and turf.

### 2.2. Croatia

**Figure 3:**
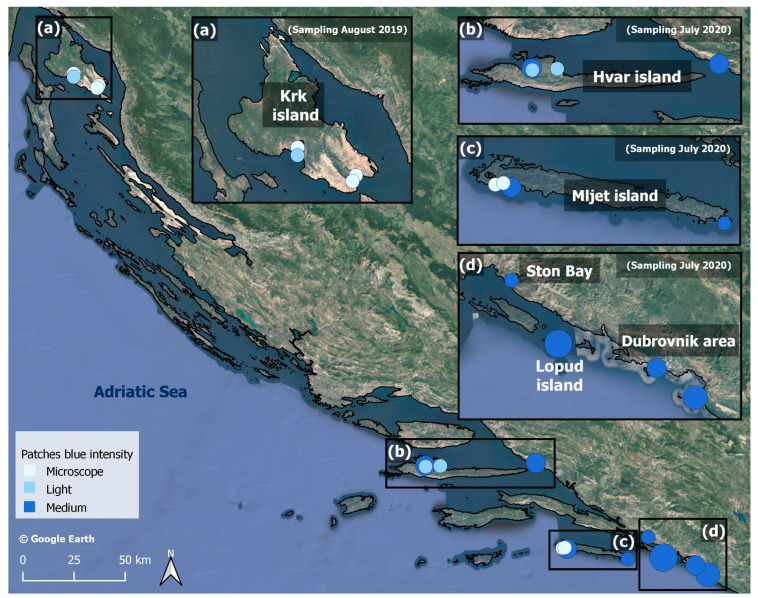
Observations of blue *Haslea* blooms, (**a**) in Krk island in August 2019 and (**b**–**d**) along the Dalmatian coast in July 2020. The blue intensity (see legend) refers to the density of the *Haslea* population, as illustrated in Figure 2.

Small blue patches were observed on Krk Island in late August of 2019. During the sampling campaign from 17th to 26th of July 2020, patches were recorded along the coast from Dubrovnik to Hvar Island (Figure 3). The site where the maximum density was observed was in Šunj beach, Lopud Island and the southern Adriatic (Figure 3d). Along the Dalmatian coast, blooms were localized on the thallus of *Padina* sp. (Phaeophyceae).

### 2.3. General Bloom Observations

Overall, blooms occurred in shallow, sunny and calm waters. Zones with the more intense blue were observed at a depth of around four meters on the non-exposed side of underwater dorsals.

**Figure 4:**
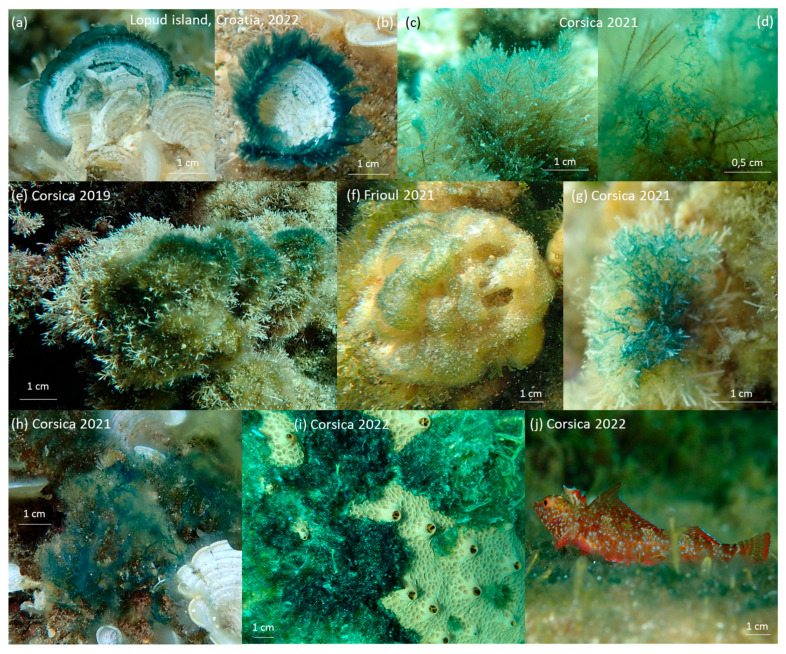
Diversity of blue *Haslea* spp. patches on different substrates: (**a**,**b**) *Padina* sp., (**c**,**d**) *Halopteris scoparia*, (**e**) *Jania* sp., (**f**) *Colpomenia sinusoa*, (**g**) *Liagora viscida*, (**h**) filamentous algae, (**i**) *Hemimycale mediterranea* (*Porifera*), (**j**) *Tripterygion* sp. (Chordata).

Thus far, *Haslea* blooms have been observed on Ochrophyta and Rhodophyta but not on Chlorophyta. They prefer to develop on turf or *Padina* sp., and have been recorded on *Halopteris scoparia* (Phaeophyceae), *Jania* sp. (Rhodophyta), *Colpomenia sinuosa* (Phaeophyceae), *Liagora viscida* (Rhodophyta) and filamentous algae like *Nematochrysopsis marina* (Pelagophyceae) and *Acinetospora crinita* (Phaeophyceae) (Figure 4). The morphology types of these algae are characterized by wide thallus or with ramification and the presence of hairlines. *Haslea* spp. blooms have been observed close to sponges (Figure 4i), but never cover them.

**Figure 5:**
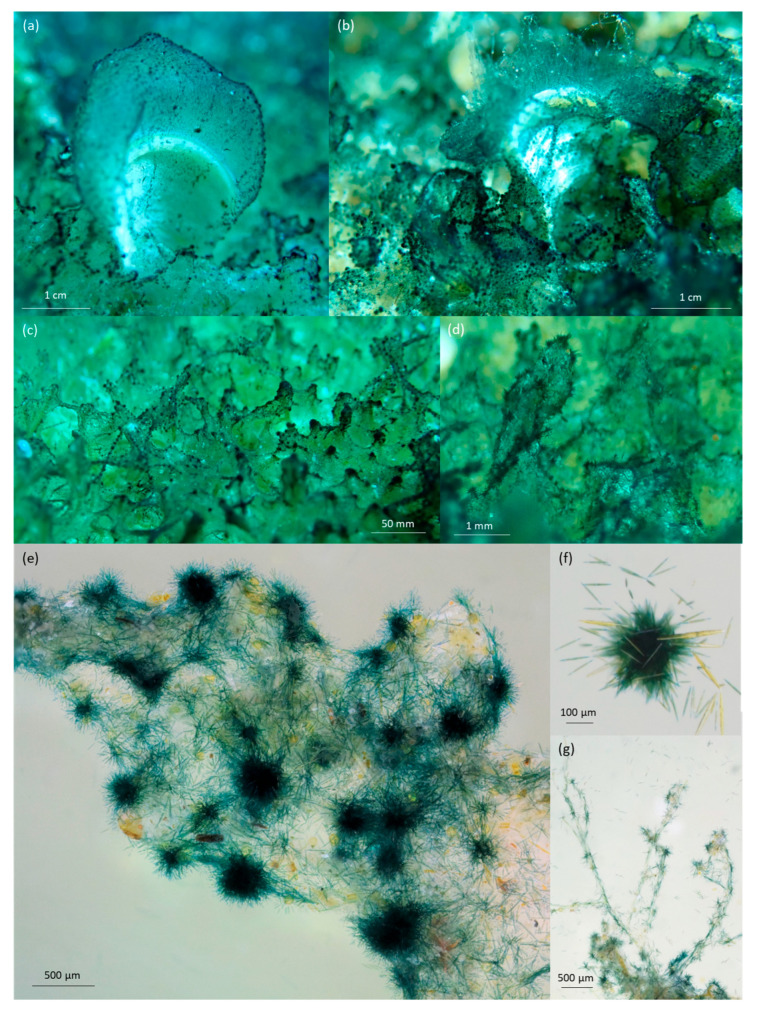
Pictures from Corsica, March 2021 (**a**–**d**). Underwater macro pictures of the blooms, covering: (**a**,**b**) *Padina* sp. with *Haslea* cells covering their hairlines and (**c**,**d**) turf; (**e**–**g**) pictures taken using binocular magnifier; (**e**) accumulation of blue *Haslea* cells on the turf biofilm; (**f**) *Haslea* accumulation forming a spiky ball; (**g**) *Haslea* cells supported by macroalgae hairlines.

Underwater macro pictures and microscope observations confirmed that the blue color of the blooms is due to an accumulation of blue *Haslea* cells trapped in the biofilm, not to the fixation of the pigment marennine on the biofilm structure itself (Figure 5). Cells gathered in the superior part of the biofilm and formed spherical structures with spike-like protrusions (Figure 5f). Blue *Haslea* cells highlight the complex arrangement of the biofilm, constructing bridges between structures of turf (Figure 5c).

### 2.4. Species of Blue Haslea Responsible for the Blooms

**Figure 6:**
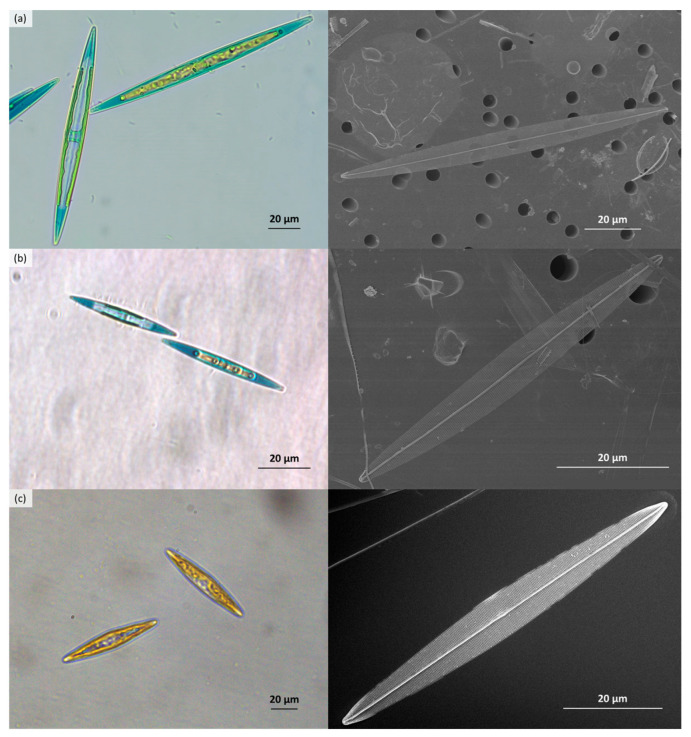
*Haslea* species found in our samples, the photos on the left are living cells observed under light microscopy (LM), while those on the right show frustules viewed under scanning electron microscopy (SEM): (**a**) *H. ostrearia*; (**b**) *H. provincialis*; (**c**) *Haslea* sp4.

**Figure 7:**
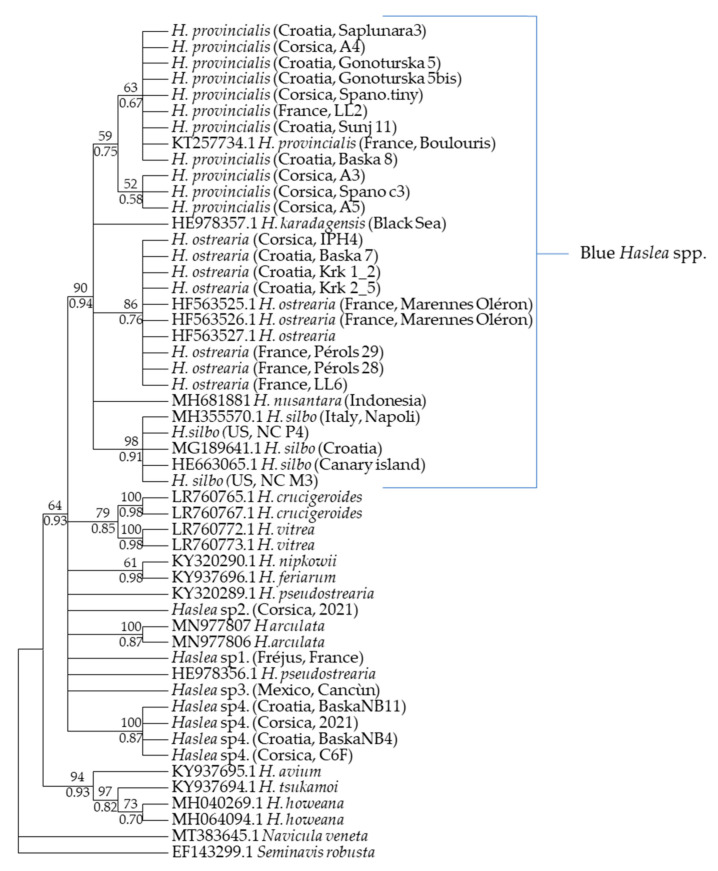
The evolutionary history of *Haslea* genus was inferred by using the maximum likelihood method and the general time reversible +G +I model (+G, parameter = 0.2834; +I, 50.72% sites) and BI method using same evolutionary model. The tree with the highest log likelihood (−1416.89) is shown. The rate variation model allowed for some sites to be evolutionarily invariable. The analysis was based on 52 rcbL sequences, which were a total of 380 positions in the final dataset. The tree was rooted with *Navicula veneta* and *Seminavis robusta*.

Blue *Haslea* species present in the blooms were identified using morphological (Figure 6) and genetic (Figure 7) approaches for each of the different survey locations. *Haslea ostrearia* was present in 2019, both in Corsican bloom and in Croatian samples. In Corsica 2021 and 2022 blooms, as well as in Croatia (2019) and in the Frioul Island (2021), several strains per location have been identified as *H. provincialis*. One non-blue species, *Haslea* sp4. (ongoing description), was also sporadically found in Corsican and Croatian samples. *Haslea* cells are lanceolate with acute apices, presenting a grid pattern on the internal view of the frustule (Figure 6).

The chloroplastic marker phylogenetic tree (Figure 7) shows that geographically close populations may be genetically distant. For instance, there is no clustering between Croatian and Corsican strains for *H. provincialis*.

### 2.5. Environmental Parameters

**Figure 8:**
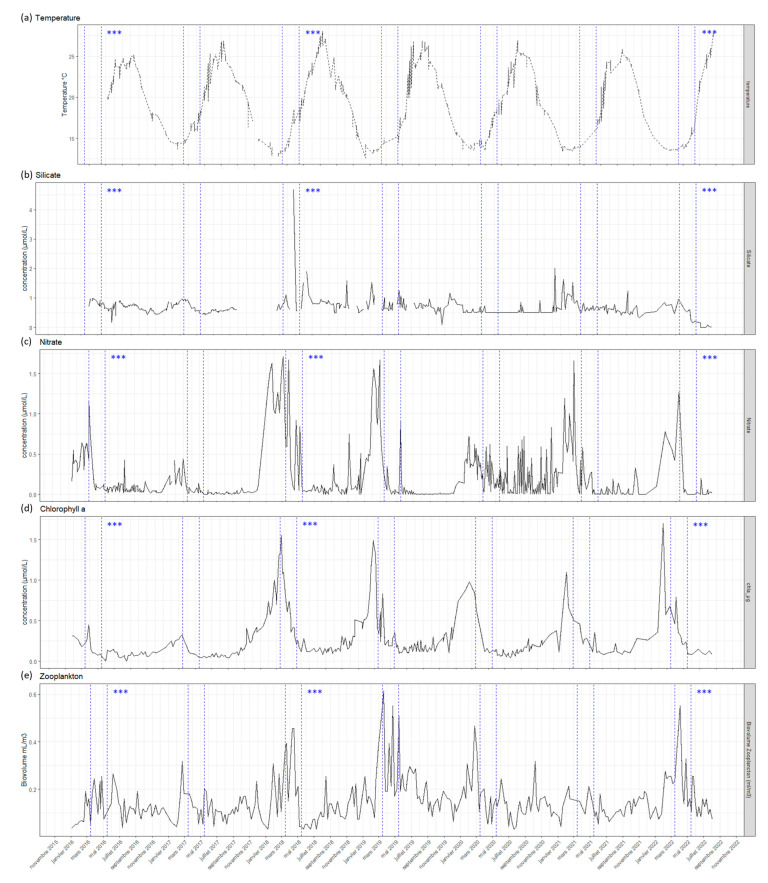
Evolution of abiotic and biotic factors over the last seven years in Calvi Bay: dashed lines represent the period of maximum blooms when the observations and sampling were made; the asterisks refer to years with intense blooms. (**a**) Temperature in °C; (**b**) silicate in µmol/L; (**c**) nitrate in µmol/L; (**d**) chlorophyll a in µg/L and (**e**) zooplankton biovolumes in mL/m^3^; dashed lines represent the maximum bloom periods; the asterisks refer to years with intense *Haslea* spp. blooms.

Different environmental parameters were examined to provide a better understanding of the global dynamics of the blue *Haslea* blooms. From 2016 to 2022, blooms of 2016, 2018 and 2022 were the most intense, the most scattered along the Revellata Cape and which lasted the longest, being observed from March to June. Variations of the environmental parameters before and after the blooms were also studied in order to identify the key ones, possibly carrying out a major role in bloom development. In Calvi Bay, over the seven last years, the temperature in the first 10 m in depth ranged from 12.6 °C (January 2019) to 28.8 °C (August 2018) (Figure 8a). When the blooms started between late February and early March, the temperature was close to its minimum, and when the blooms stopped in the beginning of June, the temperature ranged between 17.0 °C (June 2021) and 20.5 °C (June 2022). Salinity did not merely follow an annual variation with an average of 37.9 psu, with extreme values varying from 35.61 to 41.9 psu (Supplementary Materials, Figure S1). Seawater in blooming sites in Croatia was saltier and warmer when compared with sites without blooms, reaching 38.8 psu at 23.6 °C and 39.1 psu at 25.6 °C, respectively (Table 2).

The concentration of silica in seawater was investigated closely, as it could be an important factor for the development of benthic diatoms. In Calvi Bay, over the period from 2016 to 2022, silicate concentration ranged from 0.16 µM to 4.69 µM with an average of 0.72 µM. The patterns of silica concentration did not follow those of blooms, nor was there a correlation between silica concentrations and bloom intensity (Figure 8b). Along the Croatian coast, sites where the blooms were observed were not the ones with the highest silica values.

It has been noted that, in Calvi Bay, the favorable period for blooms was right after the nitrate and Chl*a* peaks (Figure 8c,d). However, the intensity of nitrate and Chl*a* peaks was not correlated with the intensity of the blooms. For example, in 2016, the *Haslea* bloom was more intense than in 2019, although 2016 peaks of nitrate and Chl*a* were lower. Nitrate concentration was constant between Croatian sites and nitrite concentration was higher in samples from blooming zones. In Gonoturska, the Chl*a* was higher than in the other Croatian locations. The phytoplankton (pelagic) blooms were followed by the zooplankton blooms (Figure 8d,e). The peaks reached their maximum in the same range as the benthic blooms of *Haslea* spp. Phosphate and ammonium concentrations did not vary much during or close to the period of blooms (Supplementary Materials, Figure S2). In Croatia, phosphate and ammonium were at their maximum in Gonoturska, at 0.4 µM and 0.6 µM, respectively, but values were more homogenous between the three other sites.

## 3. Discussion

The objective of this study was to describe *Haslea* spp. blooms in Mediterranean natural and open environments based on observations made at several time periods, in two different geographic areas, and make a comparison with previously described blue *Haslea* blooms in oyster ponds. Two open environments were chosen to study these blooms, one in Calvi Bay, Corsica, France and one in the Adriatic coast, Croatia. Blue *Haslea* blooms are characterized by an intense emerald color spreading on turf and on macroalgae like *Padina* sp. In the Ligurian Sea (Calvi Bay), blooms usually begin in late February/early March, reaching their maximum intensity and coverage during April and beginning their decrease in June. In the Adriatic Sea (Croatia), blooms were observed during July and August.

In laboratory conditions (e.g., *Haslea* culture in Erlenmeyers) or in semi-closed environments (e.g., oyster ponds), the resulting green color of cultures is partly due to the accumulation of extracellular marennine in the supernatant [21]. However, in open environments, the intensity of the blue color is a result of the accumulation of *Haslea* cells containing intracellular marennine in the benthic biofilm structure. Extracellular marennine does not seem to fix and accumulate in these benthic biofilms, but is likely diluted in the water column. *Padina* sp. constitute a substrate propitious to the development of such biofilms, because their hairlines can be used as a support and they release few allelopathic compounds [26].

Molecular analysis used to identify *Haslea* species in our samples demonstrated the presence of two blue species that were both dominant, *H. ostrearia* and *H. provincialis*, and a minority of one non-blue species. These species were observed either in the same site at different times, in the same site simultaneously or individually in one site. Different species of *Haslea* were already identified at the same time in equivalent ecological niches, for example, in the Canary Islands [27] and in Guam Islands [28]. The presence of morphologically similar but distinct benthic species in the same environment could illustrate the “paradox of the plankton” [29]. This theory was put forward because ecological models could not explain the large diversity of microorganisms inhabiting similar ecological niches in the water column. However, this theory has since been discussed and the concept of ecological niche has been redefined by taking into account the impact of population structure, predation, mutualism, abiotic stresses and bioengineering [30].

A more in-depth analysis of our data revealed that only one species of blue *Haslea* is predominant in the Mediterranean blooms: *H. provincialis*. Indeed, *H. ostrearia* can frequently be observed during the blooms, but it does not proliferate as *H. provincialis*. This biodiversity in blue *Haslea* is intriguing, but it does not concern blue species only. Previous prospections along the French coasts of the Ligurian Sea allowed us to identify various strains of both blue and non-blue *Haslea* in non-blooming conditions. For instance, in the pond named ‘’Etang de l’Or”, Hérault (France), a zone known for shellfish farming, the presence of *H. ostrearia* has been determined (unpublished). Along the French Riviera coast of La Londes-les-Maures, two strains of blue *Haslea* were identified in the same zone, *H. provincialis* and *H. ostrearia*. The blue *H. silbo* has also been previously recorded in the Mediterranean Sea, in Napoli Bay and in the Adriatic Sea. These species can be transported by currents as their life cycle could display tychopelagic phases [31]. In addition, due to their close link with aquaculture, it is worth mentioning that oysters can also be a vector of transport for microalgae [32].

The triggering factors and the dynamics of benthic blue *Haslea* blooms in the Mediterranean Sea were also investigated by examining changes in abiotic environmental conditions (nutrients, temperature, salinity) and biotic factors (Chl*a*, zooplankton) in the water column. The analysis of abiotic parameters over the last seven years in Calvi Bay showed that the timing of *Haslea* blooms follow the bloom phytoplankton. In a previous study [33], it has shown that the principal drivers of the spring phytoplankton blooms (starting in February) in Calvi Bay were the strong wind events from the previous winter. In fact, Calvi Bay is connected to a deep sea canyon (from 100 m to 1000 m deep) [34], and during strong winter wind events, an upwelling results in deep sea nutrients up to the surface due to the vertical mixing of the water column [33]. It results in an increase in nitrate and nitrite concentrations in the upper layers, which favors phytoplankton development. The end of the phytoplankton bloom is correlated with the increase in grazing pressure from the zooplankton, usually characterized by a spring peak (increase in both abundance and biovolume) taking place at the end of March [35]. In the Adriatic Sea (e.g., see [2]), phytoplankton blooms also follow a seasonal cycle, with a maximum in spring (May), a decrease through the summer months and a second increase in autumn, with phytoplankton abundance reaching its minimum during winter [36,37]. By blooming after the phytoplankton bloom, *Haslea* cells can benefit from an increase in light available through the water column, reaching the benthic environment. Moreover, as the blue *Haslea* blooms were observed in shallow waters and exposed to high irradiance, light seemed to be a key factor for species development, as it has already been found to influence growth and marennine production in laboratory conditions [18,19]. In addition, after the phytoplankton bloom, part of the organic matter escapes from the zooplankton and sinks, being eventually remineralized by the bacterial community of the benthic biofilm and then reutilized by the autotrophs. Such processes, may ultimately promote blue *Haslea* development.

Regarding salinity, the small interannual and seasonal variations suggest a weak influence of salinity on bloom evolution. Concordantly, previous observations showed that *H. ostrearia* is a euryhaline organism, as demonstrated in oyster ponds, where the salinity fluctuates due to their filling with either tide or river. This euryhaline feature has also been demonstrated in laboratory conditions [14,38].

Marine current circulation could also be a factor favoring blue *Haslea* blooms along the Revellata Cape. Indeed, in low wind conditions, subsurface currents create a small gyre on the eastern part of the Revellata Cape [39], favoring the growth and the accumulation of *Haslea* cells (Supplementary Materials, Figure S3).

Concerning the decrease and the end of blue *Haslea* blooms, it appears to be correlated with an increase in the surface water temperature. Indeed, this could be directly deleterious to blue *Haslea* cells by affecting biochemical and physicochemical processes, thus making them less competitive. No data are available regarding the thermal preferendum of *H. provincialis*; however, the optimal growth of *H. ostrearia* has been recorded at 20 °C in a laboratory [40]. In Calvi Bay, the temperature of 20 °C is usually reached by early June. The end of the bloom could also be the indirect consequence of an increase in temperature, which can alter ecosystem functioning. For instance, an increase in temperature favors diatoms with a higher thermal preferendum [41]. Moreover, a rise in temperature also enhances bacterial metabolisms, which might induce changes in the community, with the possible occurrence of bacteria that could be deleterious to *Haslea* cells [42,43]. The possible interactions between bacteria, blue *Haslea* spp. and other phytoplankton will be the subject of a future work dealing with the impacts of *Haslea* blooms on the epiphytic biofilm communities.

Strong wind events resulting in a heavy swell can also have deleterious effects on blue *Haslea* bloom development. In fact, heavy swell mixes the water column and especially affects shallow environments, leading to the disaggregation and the resuspension of the biofilm formed during the bloom. Incidentally, this mixing of waters and resuspension of cells from the biofilm can explain “tychopelagic” observations of blue *Haslea* cells in the water column. If these unfavorable conditions continue, the blue *Haslea* bloom might ultimately disappear. Such a phenomenon was observed in April of 2019, when a stormy weather was followed by a rapid and strong decline in blue *Haslea* populations, which did not recover as no sign of biofilm reformation was visible by May of the same year (Supplementary Materials, Figure S4).

Although the study of virus communities associated with the benthic biofilms was out of the scope of this work, it cannot be discarded that viruses infecting marine microalgae could have an influence on the *Haslea* bloom dynamics. According to literature [44], the more abundant a species, the more likely it can interact with a virus, meaning that viruses can play a key role in controlling populations and maintaining a high biodiversity. The interactions between viruses and microalgae have been studied in phytoplankton communities and the related results showed that, for example, *Sundstroemia setigera* (Brightwell) Medlin (formerly *Rhizosolenia setigera* Brightwell; Coscinodiscophyceae) and *Chaetoceros socialis* H.S. Lauder (Mediophyceae) viruses could induce cell lysis and therefore stop the bloom development [45,46]. In addition, it has been highlighted that vibriobenthos may display seasonal variations, for instance, in Chesapeake Bay sediments, where virus abundance was at its maximum by the end of spring and the beginning of summer, and was correlated with bacterial abundance [47,48]. Further studies could thus be conducted on *Haslea* blooms, with a focus on the variations of the viral community to determine if it plays a role in bloom dynamics.

Lastly, the comparison between blue *Haslea* blooms in the Mediterranean Sea and the ones previously described in oyster ponds in the Atlantic Ocean of western France provides a few clues for the understanding of bloom development in open natural environments, as both types of environments are highly different. Oyster ponds are closed, shallow environments and are therefore conducive to rapid variations in some forcing factors such as salinity, which can vary according to the inflow of seawater via the high tides, the inflow of fresh water from the rivers irrigating these ponds or the rainfall regime. Unlike the Atlantic Ocean, the Mediterranean Sea is considered an oligotrophic environment. The occurrence of the Atlantic and Mediterranean blooms is not the same, as they mainly take place in autumn and spring, respectively; therefore, temperature optimums are different in these two areas.

## 4. Materials and Methods

### 4.1. Choice of Substrates

- **Turf**

The term turf can be used in both terrestrial and marine environments. In this study, the term turf corresponds to the definition given by [49]—in other words, an algal mat of a few mm to cm high, covering rocky substrates. This assemblage, in the Mediterranean Sea, is composed mainly of small branches of *Halopteris scoparia* (Linnaeus) Sauvageau, small calcareous and filamentous algae. Turf also retains sediments and forms the basis of marine ecosystems, allowing numerous macroalgae to develop.

- ***Padina* sp.**

Part of the Dictyotales family, the *Padina* Adanson genus has the distinctive feature of comprising calcifying species. These brown algae have an erect complanate or flabellate thallus, up to 20 cm long. The thallus is annual, but the spike remains attached to the substrate. These photophilic algae have concentric calcified streaks. Absorbent hairs can be seen on the upper and inner parts of the thallus, increasing the surface area available for exchange with the environment. This genus is widely distributed in tropical and temperate seas [50]. In this manuscript, we refer to *Padina* sp. and not to *Padina pavonica* (Linnaeus) Thivy (the main species in the Mediterranean) because the different species of the genus are very similar morphologically and require molecular identification.

### 4.2. Study Sites

Two sites were chosen in the Mediterranean Sea to study and compare blue *Haslea* blooms. These two sites are oligotrophic environments and are considered as non-polluted areas.

**Figure 9:**
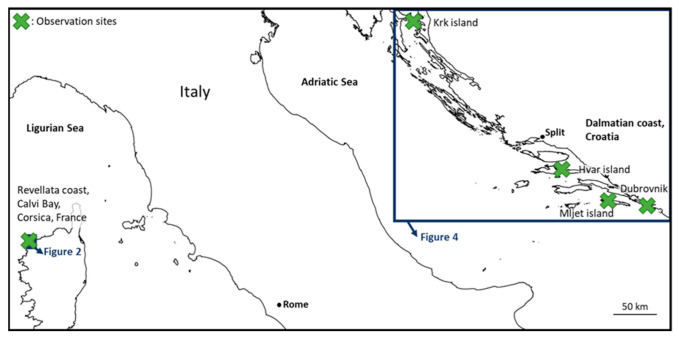
Study areas of the different observation sites in the western Mediterranean Sea.

In France, blooms were monitored along the Revellata Cape (42°34′50.8″ N, 8°43′30.8″ E), along almost 1.2 km, in the Calvi Bay, Corsica (Figure 9). The Calvi Bay has an area of 22 km^2^ and is located on the northwestern coast of Corsica Island and opens to the Ligurian Sea. Sampling campaigns were realized during springtime between the end of February and June (subdivided into three ten-day periods) from 2019 to 2022 (no data in 2020 due to the global pandemic). Previous data also indicated that blooms in 2016 and 2018 were very intense and spread all along the coast.

In addition to this area, another bloom was observed in the Ligurian Sea, in the Frioul Islands, Marseille, France. It was recorded on 6 June 2021, where pictures were taken and samples were collected to identify the blue *Haslea* species.

In Croatia, one campaign was completed in August 2019 (2 days) on the Krk Island (North Adriatic) and a second campaign took place in late July 2020 (10 days) along the Dalmatian coast, between Hvar Island and Dubrovnik (Figure 9). *Haslea* cells were isolated from both of these campaigns for identification and diatom samples were collected in 2020 to study the communities. On the island of Hvar (Central Adriatic), samples were collected from the Bay of Stari Grad (43°10′54″ N, 16°35′00″ E). In the Southern Adriatic Sea, samples were taken in “Veliko Jezero” and in “Malo Jezero” together with Gonoturska Bay (approximately 42°45′ N, 17°23′ E) that are located on the western part of Mljet Island and are part of National Park Mljet. The “Veliko Jezero” (VJ) has a surface area of 1.45 km^2^ and maximum depth of 46 m; the “Malo Jezero” (MJ) has a surface area of 0.25 km^2^ and maximum depth of 29 m. Narrow and shallow straits (from the outer sea to “VJ”: 10 m wide, 2.5 m deep; from “VJ” to “MJ”: 3 m wide, 0.5 m deep) connect the lakes with the outer southern Adriatic Sea [51].

At all sampling sites, observations were performed first while snorkeling to delimit the depth range and the depth at which the bloom was at its maximum. Then, transects were realized along the bathymetric line, where bloom was at its maximum, in order to follow its evolution. Patches were photographed, their surfaces and densities estimated. Benthic samples of turf and *Padina* sp. were collected in 50 mL falcon tubes in blooming and control areas, to verify the presence of blue *Haslea* cells under microscope.

### 4.3. Environmental Parameters

In Calvi Bay (France), environmental parameters were recorded at the Station de Recherches Sous-marines et Océanographiques (STARESO). Salinity, temperature and turbidity were measured using a CTD (conductivity–temperature–depth) sensor (EXO 2, YSI). Data from the surface to a depth of 10 m, from 2016 to 2022, were analyzed for the present work. Sampling was performed at 1 m depth for nutrients and chlorophyll *a* (Chl*a*). Silicate (SiO_4_), phosphate (PO_4_), ammonium (NH_4_), nitrate (NO_3_) and nitrite (NO_2_) were analyzed using a SAN-Skalar [52]. The concentration of Chl*a* was measured via fluorimetry using a Trilogy Turner Design Fluorimeter. Chl*a* was used as a proxy for phytoplankton biomass. Zooplankton biomass was also studied, where sub-surface horizontal hauls were taken at 3 m depth using a WP2 net of 200 μm mesh size, with a 60 cm opening diameter. Their biovolumes were determined using a graduated cylinder. When available, data were analyzed since 2016. Dashed lines represent the most favorable periods for blooms, from March to May.

For the Hvar and Mljet islands (Croatia), salinity and temperature were measured using a WTW Multiline P4 multiparametric sensor. Water samples were collected to measure silicate, phosphate, ammonium, nitrite, nitrate, total inorganic nitrogen (TIN = NH_4_ + NO_3_ + NO_2_) and Chl*a*. Nutrients were analyzed via colorimetry [52,53] and chl*a* was determined via fluorescence measurements using a Turner TD-700 Laboratory Fluorometer (Sunnyvale, CA, USA) calibrated with pure Chl*a* (Sigma-Aldrich, St. Louis, MO, USA).

### 4.4. Algal Culture, Microscopy and DNA Identification

Blue *Haslea* cells were isolated using glass Pasteur pipettes, cultured in filtered seawater and then transferred in artificial seawater medium. This medium used was made from Instant Ocean (Aquarium Systems, Sarrebourg, France) powder enriched with silicates, phosphates, nitrates, metals and vitamins. Three successive steps of re-isolation were performed before cultures were considered monoclonal. Cultures were grown and maintained in Erlenmeyer flasks under controlled conditions: temperature of 16 ± 1 °C, irradiance of 400 µmol photons/m^2^/s, with 14 h:10 h light/dark photoperiod, at Le Mans University. Irradiance was provided using fluorescent tubes (Philips TLD 36W/965) and measured using a Li-Cor LI-189 quantum meter coupled with a 2P Li-Cor Q21284 quantum sensor.

LM pictures were taken using Zeiss Axio Scope A1 and a Zeiss Axio Cam ICc5 camera. For SEM, frustules were dried onto polycarbonate membrane filters, Whatman Nuclepore with 1 μm pores, and then mounted onto aluminum stubs and sputter-coated with 20 nm of gold or gold–palladium alloy using a Q150T coater from Quorum Technologies (Laughton, UK). Observations were conducted using the ultra-high resolution scanning microscope SU8020 from Hitachi (Tokyo, Japan), from the University of Szczecin (Poland).

When culture reached 5:10^5^ cells/mL, 50 mL of the culture were centrifuged at 3500× *g* for 15 min. DNA extraction was performed on the pellet, using Plant II Nucleospin MN© kit and following manufacturer instructions. DNA quantity and quality were assessed using Nanodrop 2000 (Thermo Fisher Scientific, Waltham, MA, USA). The chloroplastic marker *rbc*L was amplified using primers *rbc*L-forward (GACCGTTACGAATCTGGTG) and *rbc*L-reverse (GACCACATGTTTTAGCAGC) for a total length of 1330 pb for each fragment. To achieve a good yield, 35 cycles of amplification were realized: denaturing for 1 min at 94 °C, annealing for 1 min at 54 °C, and an extension was conducted for 1.5 min at 72 °C. PCR products were sent to Azenta genomics for sequencing.

### 4.5. Data Analysis

Maps were conducted on QGIS software (QGIS Geographic Information System. Open Source Geospatial Foundation, version 3.16), using aerial orthophotography from IGN2017 and SHOM/IGN histolitt v2 2013 for Corsica and using Google Satellite, Map data 2015 and EEA coastline 2017 for Croatia.

The phylogenetic tree was obtained using our PCR amplification of the chloroplast marker *rbc*L sequences (DNA sequences have been submitted to GenBank to obtain a registration number) and the *rbc*L sequences available on GenBank database. Phylogenetic analyses were carried out using MEGA X (Version 10.1.8; [54]) to find the best evolutionary model and PhyML (http://phylogeny.lirmm.fr (accessed on 26 October 2023)) to determine the typology of phylogenetic trees using the maximum likelihood method. A supplementary phylogenetic analysis was made to confirm the topology of ML tree. Bayesian inference was carried out using [55]. The robustness of nodes was evaluated via bootstrap (1000 replicates) [56] replicates and rooted with *Navicula veneta* and *Seminavis robusta*.

Statistical analyses were performed using R software (version 4.0.3), and plots were created using the ggplot2 package.

## 5. Conclusions

The present work is the first investigation on the development of blue *Haslea* blooms in natural marine open environments. Blooms of *H. ostrearia* have long been observed in autumn and to a lesser extent in spring, in oyster ponds along the French Atlantic coast, which are closed anthropized environments. In the Mediterranean Sea, blue *Haslea* spp. blooms occur in open environments. Species involved in both types of blooms are different; *H. ostrearia* is responsible for blooms in oyster ponds: it has been shown to be cosmopolite and has been observed in the northern part of the Mediterranean Sea. *Haslea provincialis* seems rather endemic and appears to be the main species responsible for the blooms in the Mediterranean Sea. These two species can thus have distinct preferenda. This study allowed us to better understand the environmental parameters related to blue *Haslea* spp. bloom development in open environments. A temporal lag was noticed between the Ligurian Sea and the Adriatic Sea—blooms occurring in spring and summer, respectively. In both cases, they were observed after the planktonic bloom. It can thus be hypothesized that the benthic bloom of blue *Haslea* spp. benefits from a better access to light. Water currents, for instance, heavy swell, can mechanically disaggregate the biofilm, but temperature might also, directly or indirectly, have an impact on the decrease in *Haslea* blooms due to a possible shift in the microorganism communities of the biofilm. The present study contributes to the improvement of our understanding of bloom dynamics in phototrophic biofilms.

## Figures and Tables

**Table 1:** Percentage of blue *Haslea* cells in the total diatom community, calculated in fresh fractions (N = 125). Comparison between the color of patches and the kind of substrate.

	Patches Intensity (Color)					Substrate with Blue Coloration	
	Control	Tot Blue	Light	Medium	High	*Padina* sp.	Turf
Average (%)	1.0	24.6	7.3	29.1	42.8	26.7	23.2

**Table 2:** Environmental parameters recorded on Croatian sites, in July 2020.

	Sampling Site	*Haslea* Bloom	*Haslea* in Microscopy	Date	Salinity (psu)	Temperature (°C)	PO_4_ (µM)	SiO_4_ (µM)	NH_4_ (µM)	NO_2_ (µM)	NO_3_ (µM)	TIN (µM)	Chl*a* (µM)
Mljet Island National Park	Malo jezero	no	no	17 July 2020	39.2	26.00	0.126	3.370	0.301	0.017	0.241	0.560	0.18
	Veliko jezero	no	yes	17 July 2020	39.0	25.3	0.152	4.308	0.340	0.013	0.268	0.622	0.16
	Gonoturska Bay	yes	yes	17 July 2020	38.8	23.6	0.365	6.185	0.627	0.027	0.219	0.874	0.42
Hvar Island	Stari Grad Bay	yes	yes	21 July 2020	38.7	23.6	0.111	2.633	0.385	0.029	0.244	0.659	0.11

## Data Availability

The data presented in this study are available on request from the corresponding author.

## Supplementary Materials

The following supporting information can be downloaded at: https://www.mdpi.com/article/10.3390/md21110583/s1, Figure S1. Yearly salinity (PSU) evolution over the last seven years in Calvi Bay, dashes lines represent the maximum blooms period, the asterisks refer to years with intense blooms; Figure S2. Ammonium, Nitrite and Phosphate concentrations, in µmol/L, evolution over the last seven years in Calvi Bay, dashes lines represent the maximum blooms period, the asterisks refer to years with intense blooms; Figure S3. Subsurface current in Calvi Bay during low wind conditions; Figure S4. Decreased of blue Haslea bloom, (a) seasonal maximum of the bloom, (b) bloom after the strong wind and swell event and (c) ending of the bloom.
