# Supplementary material for: Benthic Diatom Blooms of Blue Haslea spp. in the Mediterranean Sea
Source: Mar Drugs. 2023 Nov 8;21(11):583. doi: 10.3390/md21110583 (PMC10672038; doi:10.3390/md21110583)
Supplement: Supplementary file 1 [file marinedrugs-21-00583-s001.zip › marinedrugs-2638898-supplementary.pdf]

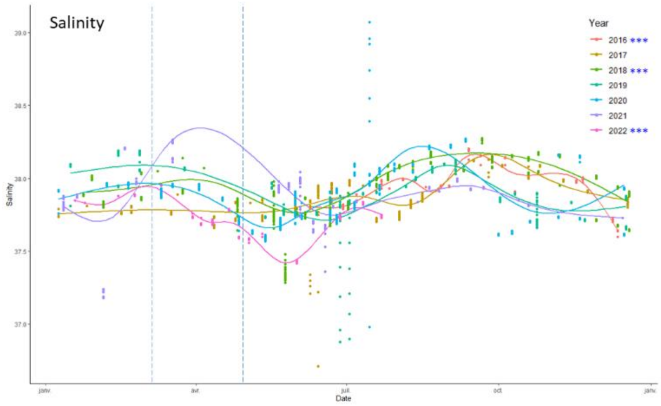

Figure S1. Yearly salinity (PSU) evolution over the last seven years in Calvi Bay, dashes lines represent the maximum blooms period, the asterisks refer to years with intense blooms.

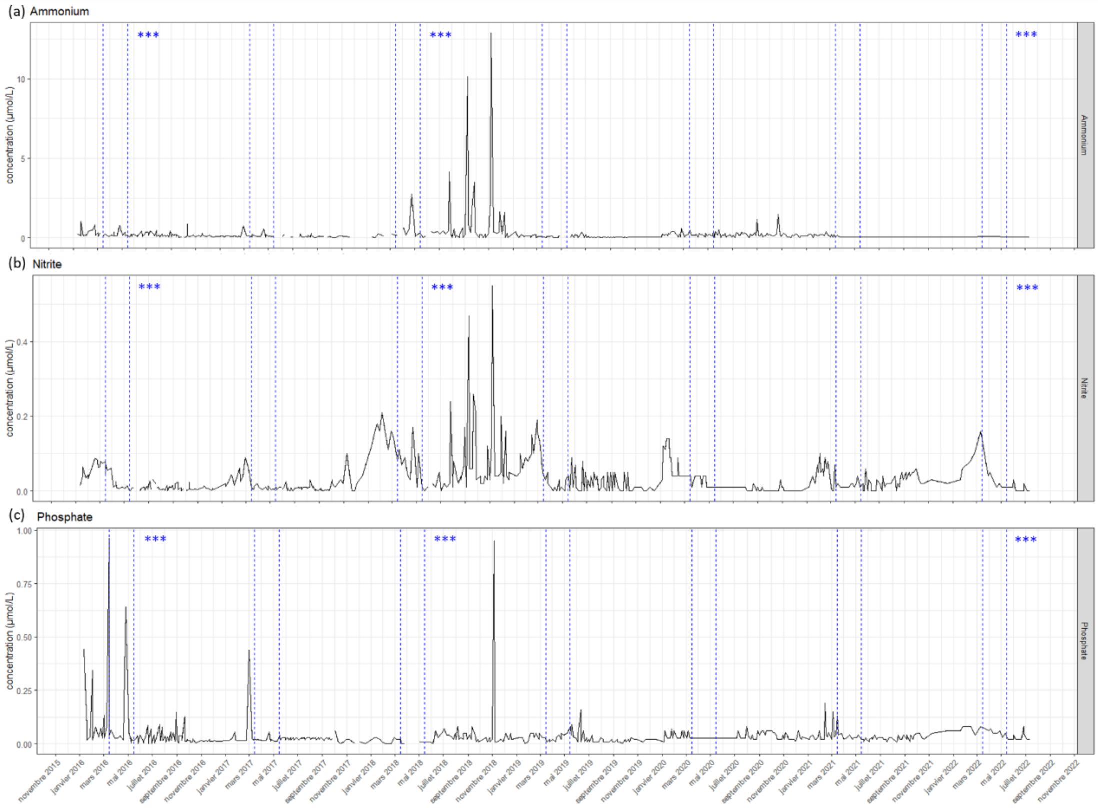

Figure S2. Ammonium, Nitrite and Phosphate concentrations, in µmol/L, evolution over the last seven years in Calvi Bay, dashes lines represent the maximum blooms period, the asterisks refer to years with intense blooms.

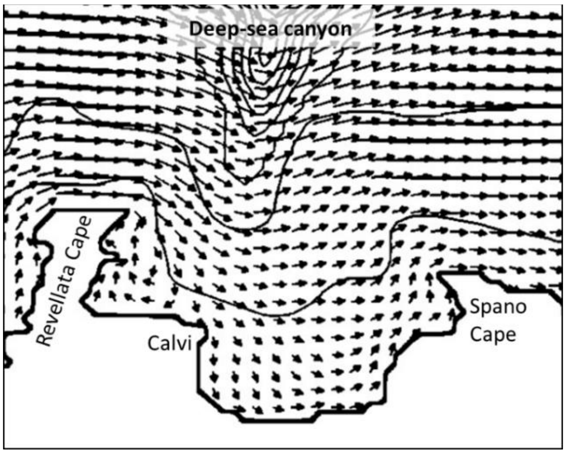

Figure S3. Subsurface current in Calvi Bay during low wind conditions (modelised by Skliris *et al*. 2001).

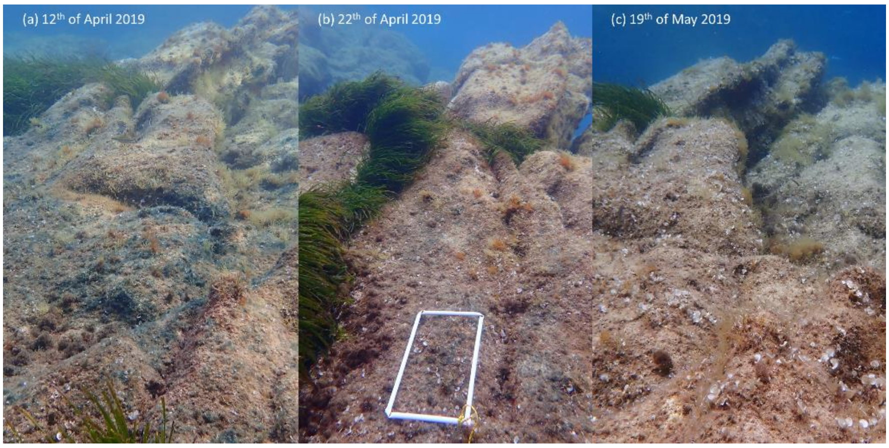

Figure S4. Decreased of blue *Haslea* bloom, a) seasonal maximum of the bloom, b) bloom after the strong wind and swell event and c) ending of the bloom.
